# Diagnostic Yield of a Targeted Next-Generation Sequencing Gene Panel for Pediatric-Onset Movement Disorders: A 3-Year Cohort Study

**DOI:** 10.3389/fgene.2019.01026

**Published:** 2019-10-29

**Authors:** Federica Graziola, Giacomo Garone, Fabrizia Stregapede, Luca Bosco, Federico Vigevano, Paolo Curatolo, Enrico Bertini, Lorena Travaglini, Alessandro Capuano

**Affiliations:** ^1^Movement Disorders Clinic, Department of Neurosciences, Bambino Gesù Children’s Hospital, Rome, Italy; ^2^Department of System Medicine, University of Rome Tor Vergata, Rome, Italy; ^3^University Hospital Pediatric Department, Bambino Gesù Children’s Hospital, University of Rome Tor Vergata, Rome, Italy; ^4^Department of Neuroscience, Unit of Neuromuscular and Neurodegenerative Disease, Bambino Gesù Children’s Hospital, Rome, Italy; ^5^Department of Sciences, Roma Tre University, Rome, Italy

**Keywords:** dystonia, chorea, neurodegeneration with brain iron accumulation disorders, genetics, next-generation sequencing, children, myoclonus, neurotransmitters

## Abstract

In recent years, genetic techniques of diagnosis have shown rapid development, resulting in a modified clinical approach to many diseases, including neurological disorders. Movement disorders, in particular those arising in childhood, pose a diagnostic challenge. First, from a purely phenomenological point of view, the correct clinical classification of signs and symptoms may be difficult and require expert evaluation. This is because the clinical picture is often a mixture of hyperkinetic and hypokinetic disorders, and within hyperkinetic movement disorders, combined phenotypes are not unusual. Second, although several genes that cause movement disorders in children are now well-known, many of them have only been described in adult populations or discovered in patients after many years of disease. Furthermore, diseases that alter their mechanisms from childhood to adulthood are still little known, and many phenotypes in children are the result of a disruption of normal neurodevelopment. High-throughput gene screening addresses these difficulties and has modified the approach to genetic diagnosis. In the exome-sequencing era, customized genetic panels now offer the ability to perform fast and low-cost screening of the genes commonly involved in the pathogenesis of the disease. Here, we describe a 3-year study using a customized gene panel for pediatric-onset movement disorders in a selected cohort of children and adolescents. We report a satisfying diagnostic yield, further confirming the usefulness of gene panel analysis.

## Introduction

Movement disorders (MDs) are a heterogeneous group of neurological conditions characterized by the production of abnormal voluntary or involuntary movements (Sanger et al., 2010). In children, MDs frequently occur in complex presentations, with different MDs appearing simultaneously or sequentially in the same patient. Frequently, MDs can coexist with other neurological disorders, resulting in complex neurodevelopmental conditions (Cordeiro et al., 2018). A systematic approach is needed to reach the correct diagnosis, and the first step usually relies on the phenomenological classification of the disorder (Sanger, 2003; Abdo et al., 2010; O’Malley and Gilbert, 2018).

Many pediatric-onset MDs are monogenic diseases, and their genetic landscape has been widely explored in recent years due to the introduction and spread of next-generation sequencing (NGS) technology. This has had a significant impact on the definition of phenotypes and syndromes. NGS denotes a group of technologies that allow the sequencing of a large amount of nucleic acid, representing an entirely new paradigm in sequencing technology that follows Sanger sequencing (Sanger et al., 1977; Lohmann and Klein, 2014). Using targeted resequencing, a subset of regions of interest on the genome can be sequenced by NGS. This strategy is widely used to sequence selected genes involved in the pathogenesis of specific disease (Reale et al., 2018). Few studies have investigated the diagnostic yield of gene panel analysis in MDs (van Egmond et al., 2017; Montaut et al., 2018; Reale et al., 2018), and molecular investigations in pediatric-onset MDs are highly variable, according to the availability of local resources and the methods of molecular genetics laboratories.

In this study, we retrospectively analyzed the diagnostic performance of a customized, targeted NGS panel specifically designed for pediatric-onset MDs in a large cohort of children referred to our institution.

## Materials and Methods

### Participants

This retrospective study was conducted in a cohort of pediatric patients referred to the Movement Disorders Clinic of Bambino Gesù Children’s Hospital in Rome, a tertiary referral hospital for rare neurological diseases in children. All patients underwent genetic tests for MDs with the customized NGS panel available at the molecular medicine laboratory in our department. All data available from January 2015 to November 2018 were collected. Electronic patient files were reviewed by pediatric neurologists experienced in MDs, and the phenomenology of the MDs was classified by reviewing video-recorded examinations. Furthermore, the clinical features of MDs, neuroimaging tests, biochemical work-up, and neurophysiological tests (when performed) were reviewed. Inclusion criteria were diagnosis of an MD (including dystonia, chorea, myoclonus, and tremor) with onset before 18 years of age and/or positive family history of an MD and ruling out of secondary causes (such as perinatal asphyxia or cerebral infection) during the diagnostic work-up. Patients with tic disorders or Tourette’s syndrome, pure cerebellar ataxia, or hereditary spastic paraplegia were excluded, as were patients with incomplete medical records. In addition, patients who underwent the same gene panel analysis for a clinical suspicion of familial hemiplegic migraine (fHM) or childhood periodic syndromes (e.g., paroxysmal torticollis) were not included in the analysis.

The patients were grouped into five categories according to their most prominent MD: isolated dystonia, combined dystonia (when dystonia was associated with other MDs), paroxysmal MDs (PMDs), chorea, and tremor. The PMD group was divided into subgroups using phenomenology and the trigger factors of the attacks. DNA samples of index patients and both parents (trios) were collected from peripheral blood leukocytes using standard procedures. Written informed consent for genetic testing was obtained from patients’ legal guardians or directly from the patient for those aged 18 years or older. During the diagnostic work-up, all patients underwent brain Magnetic Resonance Imaging (MRI), and neurophysiological exams, neuropsychological tests, and biochemical studies on blood, urine, and cerebrospinal fluid were performed following a case-by-case clinical assessment.

### Targeted Sequencing

Targeted sequencing of index cases’ DNA was performed using a mean of a customized panel (Nextera Rapid Custom Enrichment, Illumina, San Diego, CA), including 102 genes, selected in accordance with the results of a systematic literature review of studies of MD-associated genes. The panel was designed with the Illumina Design Studio tool. The region of interest was the coding sequence of each gene with a ±20 bp intronic flanking region to include splicing mutations. The 3′ and 5′ untranslated regions (UTRs) were included in the sequenced region only in genes with previously described pathogenic variants in the UTRs. Gene libraries were obtained from a Nextera Rapid Capture Target Enrichment Kit and sequenced on a *MiSeq* platform (Illumina). The generated reads were aligned to human genome assembly hg38 (December 2013, GRCh38). Variants were called using the HaplotypeCaller tool of GATK software, version 4.3 (Cambridge, MA, USA) and annotated with the ANNOVAR software tool (Wang et al., 2010). Annotated data were filtered to exclude intronic and synonymous variants that were not predicted to affect splice sites, as well as variants with reported minor allele frequency (MAF) ≥ 0.01 in publicly available resources on human variation, such as dbSNP ver. 144, 1000 Genomes, Exome Aggregation Consortium (ExAC), NHLBI Exome Sequencing Project Exome Variant Server (EVS). Missense variants of suspected pathogenicity were investigated using *in silico* prediction tools, including PolyPhen-2 (http://genetics.bwh.harvard.edu/pph2/), SIFT (http://sift.jcvi.org/), MutationTaster (http://www.mutationtaster.org/), and Alamut (https://www.interactive-biosoftware.com/). Changes affecting the splice site were investigated with Human Splicing Finder (http://www.umd.be/HSF/). Novel variants were considered to be variants of unknown significance when these were rare (MAF < 0.01%) in population databases and/or were predicted not to be pathogenic by prediction tools. Putative pathogenetic variants were validated by Sanger sequencing and investigated in the parents to assess intrafamilial segregation. Possible copy number variations were investigated using multiplex ligation-dependent probe amplification or real-time PCR techniques.

## Results

### Clinical Characterization of the Cohort

**Figure 1:**
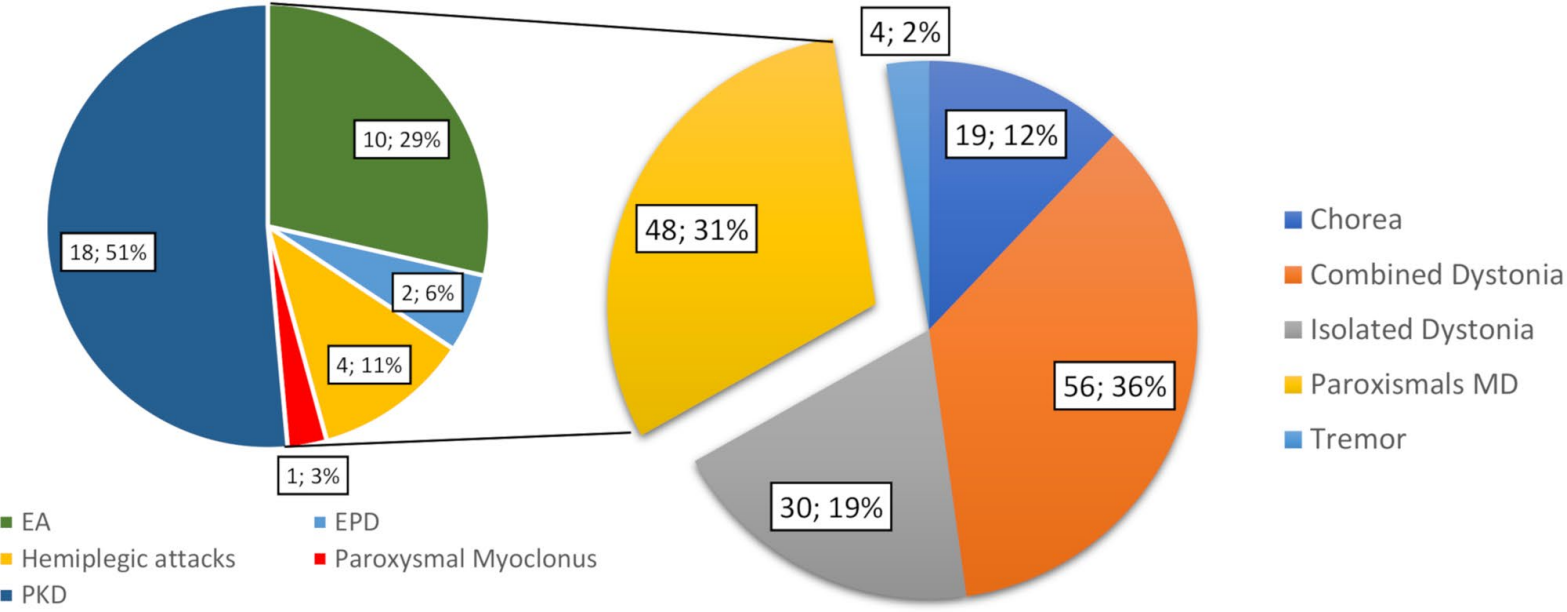
Number and percentage of the total patients by classification according to prominent MD phenomenology. Following the phenomenology of the attacks, the paroxysmal MD group is further classified into episodic ataxia (EA), paroxysmal kinesigenic dyskinesia (PKD), and exercise-induced paroxysmal dyskinesia (EPD), hemiplegic attacks, and paroxysmal myoclonus groups. MD, movement disorder.

**Figure 2:**
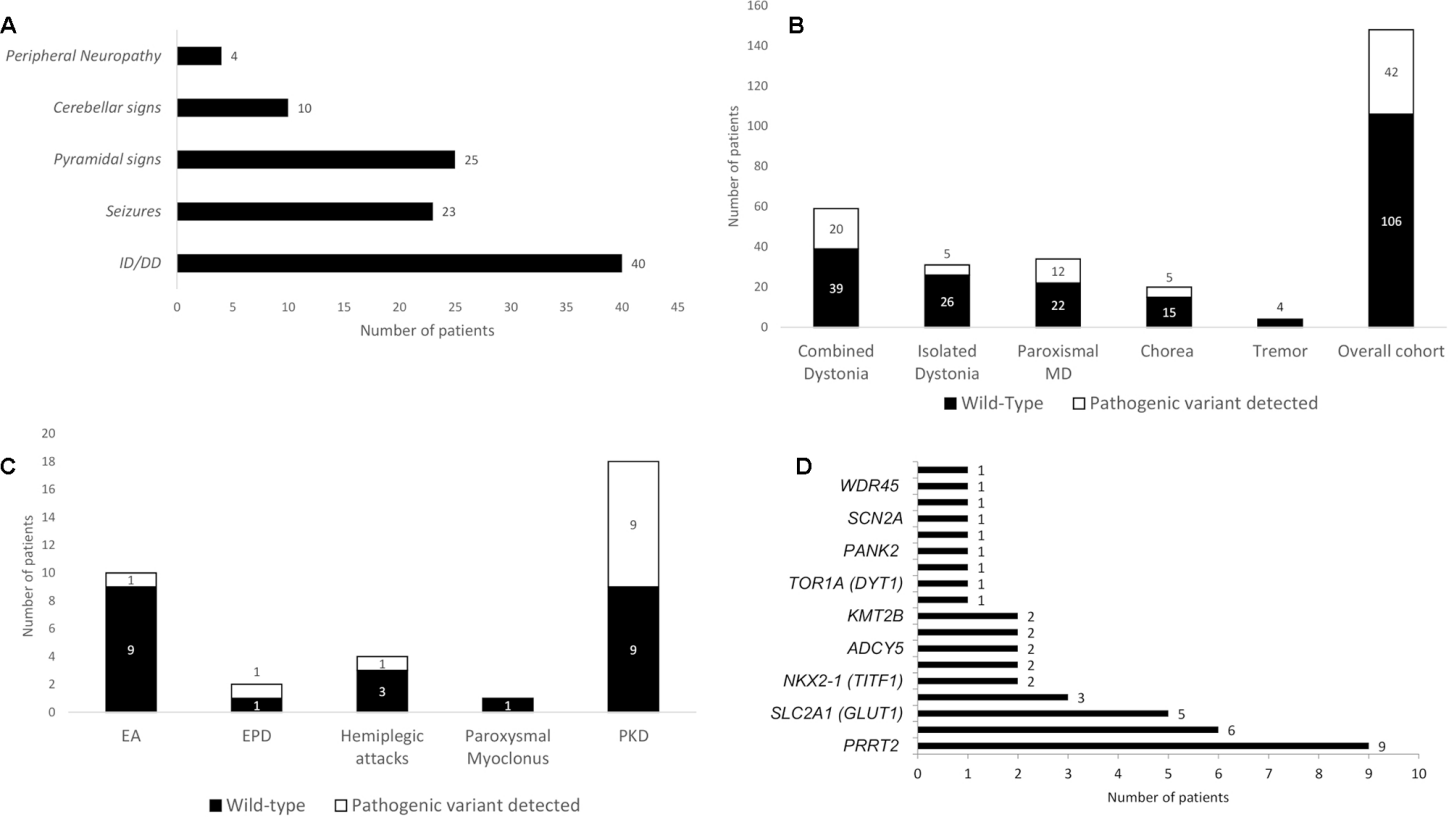
Associated neurological features of the cohort and diagnostic yield of the gene panel analysis. **(A)** Associated neurological features in the overall cohort. **(B)** Diagnostic yield in the overall cohort and in the different groups, expressed as total number of patients receiving a definite molecular diagnosis through gene panel analysis. **(C)** Diagnostic yield of gene panel analysis in paroxysmal MD according with their phenomenological classification. **(D)** Distribution of pathogenetic variants encountered per gene (see **Appendix 2** in the **Supplementary Materials** for the complete list of genes sequenced in the panel). DD, developmental delay; EA, episodic ataxia; EPD, exercise-induced paroxysmal dyskinesia; ID, intellectual disability; MD, movement disorder; PKD, paroxysmal kinesigenic dyskinesia.

Overall, 204 patients underwent gene panel analysis for MD and 38 were excluded because they were referred with a clinical diagnosis of fHM or paroxysmal torticollis. Then, 18 patient files were excluded due to incomplete chart documentation. Thus, we included 148 patients in the study, of whom 82 were male (55%) and 66 were female (45%). The median age of onset of disease was 8 (range 0–17) years old, and the median age at the time of gene panel analysis was 9.5 (range 0–20) years. A total of 134 cases (93%) were sporadic (meaning that no other relatives were affected), and the remaining 14 (7%) had a family history of disease. Of the total, 59 patients were investigated for combined dystonia (40%), 31 for isolated dystonia (21%), 34 patients for (PMDs) (23%), 20 for chorea (13%), and 4 for tremor (3%) (**Figure 1**). Among the 34 patients referred for PMD, 10 were investigated for episodic ataxia (EA), 18 for paroxysmal kinesigenic dyskinesia (PKD), 2 for exercise-induced paroxysmal dyskinesia (EPD), 4 for hemiplegic attacks, and 1 for paroxysmal myoclonus (**Figure 1**). In all, 102 patients (68.9%) presented neurological features other than MDs (**Figure 2A**).

### Diagnostic Yield

Pathogenic variants were detected in 42 out of 148 patients, leading to a diagnostic yield of 28% (**Table 1** in the **Supplementary Materials**), and 106 patients were left without a definite molecular diagnosis after gene panel analysis (**Figure 2B**). We detected pathogenic variants in 20 of 59 patients in the combined dystonia group (34%), 5 of 31 in the isolated dystonia group (16%), 5 of 20 in the chorea group (25%), and 12 of 34 in the pMD group (35%, **Figure 2B**). Among the pMD patients, the diagnostic rate was 50% for PKD and EPD patients, 25% for patients with hemiplegic attacks, and 10% in those with EA. No pathogenic variants were identified in the only patient with paroxysmal, non-epileptic myoclonic attacks or in the patients investigated for tremor (**Figure 2C**).

**Table 1:** All variants detected in our cohort classified on the basis of main phenotype.

Phenotype	Gene (#MIM number)	Pattern of inheritance	DNA variation	Amino acid change	Allele transmission	Segregation	Reference
**Chorea**	*NKX2.1* *(#600635)*	AD	c.344delG	p.G115AfsX9	het	Sporadic	Novel
		AD	c.391C>T	p.Q131X	het	Familial	Novel
	*ADCY5* *(#600293)*	AD	c.2083C>G	p.R695G	het	Sporadic	Novel
		AD	c.1253G>A	p.R418Q	het	Sporadic	Chen et al. (2015)
	*GNAO1 (#139311)*	AD	c.709G>A	p.E237K	het	Sporadic	Schirinzi et al., 2018a
**Dystonia**	*ADAR1* *(#606601)*	AR	c.557C>G+c.2894C>T	p.P193A+ p.P965L	comp het	Sporadic	Rice et al. (2012) Wang et al. (2010)
		AD	c.3019G>A	p.G1007R	het	Sporadic	Suzuki et al. (2005)
	*ATP1A2* *(#182340)*	AD	c.2335A>C	p.S779R	het	Sporadic	Novel
		AD	c.889G>A	p.A297T	het	Sporadic	Novel
	*ATP1A3* *(#182350)*	AD	c.2227_2229delGAC	p.D743del	het	Sporadic	Schirinzi et al., 2018b
		AD	c.2266C>T	p.R756C	het	Sporadic	Kanemasa et al. (2016)
		AD	c.2266C>T	p.R756C	het	Familial	Kanemasa et al. (2016)
		AD	c.2452G>A;	p.E818K	het	Sporadic	Demos et al. (2014)
		AD	c.2767G>A	p.D923N	het	Sporadic	Anselm et al. (2009)
		AD	c.2838G>C	p.G947R	het	Sporadic	Heinzen et al. (2012)
	*CACNA1B* *(#601012)*	AD	c.5381C>T	p.T1794M	Het	Sporadic	Novel
	*TOR1A* *(#605204)*	AD	c.907_909delGAG	p.E303del	Het	Familial	Ozelius et al. (1997)
	*GNAO1* *(#139311)*	AD	c.535A>G	p.R179G	Het	Sporadic	Novel
		AD	c.607G>A	p.G203R	Het	Sporadic	Schirinzi et al., 2018a
	*KCTD17* *(#616386)*	AD	c.508-2A>T			Sporadic	Graziola et al. (2018)
	*KMT2B* *(#606834)*	AD	c.1664dupC	p.V557GfsX4	Het	Sporadic	Novel
		AD	c.649dupC	p.R217fsX34	Het	Sporadic	Novel
	*SCN2A* *(#182390)*	AD	c.4951T>G	p.F1651V	Het	Sporadic	Novel
	*SLC2A1* *(#138140)*	AD	c.152G>A	p.R51H	Het	Familial	Novel (rs201815571)
		AD	c.458G>C	p.R153P	Het	Sporadic	Novel
	*SGCE* *(#604149)*	AD	c.386T>C	p.I129T	Het	Sporadic	Tedroff et al. (2012)
	*STXBP1* *(#602926)*	AD	c.1324A>G	p.N442D	Het	Sporadic	Novel
	*PANK2* *(#606157)*	AR	c.1151C>A+c.444_446delG	p.P384H+ p.E148fsX55	comp het	Sporadic	Novel Novel
	*PLA2G6* *(#603604)*	AR	c.607C>T+dupEx 6-7	p.Q203X+ dup6-7	comp het	Sporadic	Novel –
	*WDR45 (#300526)*	XLD	c.7C>T	p.Q3X	Het	Sporadic	Novel
**Paroxysmal MD**	*PRRT2* *(#614386)*	AD	c.577G>T	p.E193X	Het	Sporadic	Novel
		AD	c.649dupC	p.R217fsX8	Het	Sporadic	Chen et al. (2011)
		AD	c.649dupC	p.R217fsX8	Het	Familial	Chen et al. (2011)
		AD	c.649dupC	p.R217fsX8	het	Familial	Chen et al. (2011)
		AD	c.649dupC	p.R217fsX8	het	Familial	Chen et al. (2011)
		AD	c.649dupC	p.R217fsX8	het	Familial	Chen et al. (2011)
		AD	c.649dupC	p.R217fsX8	het	Familial	Chen et al. (2011)
		AD	c.649dupC	p.R217fsX8	het	Familial	Chen et al. (2011)
		AD	c.649dupC	p.R217fsX8	het	Familial	Chen et al. (2011)
	*SLC2A1* *(#138140)*	AD	c.275+3A>T	p.R92fsX26	het	Sporadic	Novel
		AD	c.972+1G>T	-	het	Sporadic	Wang et al. (2000)
		AD	c.1097_1100delATCT	p.Y366X	het	Sporadic	Novel

*AD, autosomal dominant; AR, autosomal recessive; XLD, X-linked; het, heterozygosis; comp het, compound heterozygosis.*

The distribution of the pathogenic variants detected in specific genes is shown in **Figure 2D**. A total of 28 patients exhibited *de novo* variants; in the remaining patients, autosomal dominant and autosomal recessive inheritance was found in 11 and 2 patients, respectively. One case was X-linked. Nine patients had a variant in the *PRRT2* gene, and eight of those nine carried the classical PRRT2 mutation variant (Chen et al., 2011); in the *ATP1A3*, *SLC2A1*, and *GNAO1* genes, pathogenic variants were detected six, five, and three times, respectively. *NKX2-1*, *ADAR1*, *ADCY5*, *ATP1A2*, and *KMT2B* were found to be mutated twice each, and *CACNA1B*, *TOR1A*, *KCTD17*, *PANK2*, *PLA2G6*, *SCN1A*, *SCN2A*, *STXBP1*, and *WDR45* were found to be mutated one time each. **Table 1** summarizes all pathogenic variants detected.

### Peculiar Findings

The application of gene panel analysis allowed previously known phenotypes to be expanded and new genotypes to be detected for several pediatric-onset MDs.

A *de novo*, previously unreported, heterozygous variant in the *GNAO1* gene (c.535A>G, p.Arg179Gly) was found in a male subject with a progressive upper body dystonia with onset in the second decade, first involving the arms and later spreading to the cranio-cervical region. He presented with a moderate intellectual disability and was suffering from partial epilepsy, controlled by therapy with carbamazepine. He had never experienced an episode of acute dystonia exacerbation. Taken together with the findings of a recent report (Kelly et al., 2019), this finding expands the spectrum of *GNAO1*-related neurological conditions beyond the two most frequently reported and severe phenotypes, namely, early infantile epileptic encephalopathy (EIEE17, OMIM 615473) and early-onset hyperkinetic phenotype (OMIM 617493) (Schirinzi et al., 2018a).

In addition, we detected a novel heterozygous variant in the *KCTD17* gene (c.508-2A>T) in a girl with a childhood-onset complex MD, mainly characterized by myoclonic dystonia. This variant affects the acceptor splice site of the exon 5, unveiling an alternative cryptic site 34 bp downstream and determining the skipping of the first 35 nucleotides of the exon. This results in a shift of the reading frame with a premature stop codon introduction in exon 7, which causes reduced protein expression (Graziola et al., 2018). This finding was further confirmed by an independent group that described a boy with childhood-onset myoclonus-dystonia harboring a novel *KCTD17* variant in an adjacent nucleotide (c.508-1G>C) affecting the same splice site (Marcé-Grau et al., 2019).

Finally, we found a novel, heterozygous missense variant affecting the *STXBP1* gene (c.1324A>G, p.N442D) in a 12-year-old boy with a severe neurodevelopmental disorder characterized by infantile-onset, drug-resistant epilepsy (with generalized myoclonic and focal motor seizures), an intellectual disability, and a peculiar complex MD phenotype with hypokinetic-rigid and dystonic features, with marked fluctuations in severity from day to day. Cerebrospinal fluid (CSF) neurotransmitter analysis revealed low levels of homovanillic and 5-hydroxyindoleacetic acids. First described as causative of a severe early infantile epileptic encephalopathy (EIEE4, OMIM 612164) (Saitsu et al., 2008), the *STXBP1* gene is now known to cause a severe neurodevelopmental disorder with almost constant intellectual disability, a high prevalence of epilepsy, and frequent occurrence of various MDs (Stamberger et al., 2016). This previously unreported variant falls within the 3b domain of the protein, with missense variants affecting the adjacent codon being already reported in patients with EIEE4 (Stamberger et al., 2016). Despite the numerous reports of *STXBP1*-encephalopathy, the clinical and biochemical picture of related MD is largely unknown.

## Discussion

Over the last decade, NGS techniques have increasingly been used as a diagnostic tool for rare genetic diseases (Neveling et al., 2013; van Egmond et al., 2017; Cordeiro et al., 2018; Montaut et al., 2018; Reale et al., 2018). To date, few studies have investigated the diagnostic yield of molecular testing in MD (Neveling et al., 2013; van Egmond et al., 2017; Cordeiro et al., 2018; Montaut et al., 2018; Reale et al., 2018). The overall rate of molecular diagnosis in previous studies ranges from 11% to 51%. However, the results of previous studies are not generalizable, due to heterogeneous study designs, sample sizes, inclusion criteria, and specific diagnostic techniques (Neveling et al., 2013; van Egmond et al., 2017; Cordeiro et al., 2018; Montaut et al., 2018; Reale et al., 2018). To the best of our knowledge, this is the first study to specifically address the diagnostic yield of a customized gene panel for MD in a large pediatric cohort.

Our diagnostic rate of 28% is slightly higher than the rates reported in previous studies that feature gene panel analysis for MD, where diagnostic rates have ranged from 11% to 22% (van Egmond et al., 2017; Montaut et al., 2018; Reale et al., 2018). Even if these differences can be attributed to heterogeneous cohort sizes and variant panel design, this finding suggests that the application of MD gene panels to selected target populations (such as children) can improve their diagnostic yield. Higher diagnostic rates have been reported only in studies with combinations of different genetic investigation approaches, such as direct testing, multiple gene panel analysis, and whole-exome sequencing (Cordeiro et al., 2018). A higher diagnostic rate was found in the PMD group (35%), similar to the rate reported by Montaut et al. (2018) in a smaller cohort (35% in 20 patients).

In contrast to previous reports (Montaut et al., 2018; Reale et al., 2018), a relatively high diagnostic rate was found in patients with dystonia (28%, where both isolated and combined forms are considered), probably reflecting a wider genetic landscape of onset in childhood and adolescence relative to adult-onset dystonia. In addition, the diagnostic rate in combined dystonia was considerably higher than in isolated forms of dystonia (34% vs. 16%), suggesting a higher yield for gene panel analysis in complex dystonia-plus syndromes.

A diagnostic rate of 25% was found in samples analyzed for suspicion of genetic chorea, very similar to the report of Montaut et al. (2018). By contrast, the diagnostic rate in patients investigated for tremor was null. There may be two reasons for this finding. First, the smaller sample of patients analyzed for tremor syndromes might drive this result. Second, no gene included in the panel specifically targeted isolated tremor syndromes.

A surprising result for a pediatric cohort was that no pathogenic variant was found in genes involved in the synthesis or transportation of neurotransmitters. No patient undergoing CSF neurotransmitter analysis showed a metabolite profile that could be clearly diagnosed as a primary defect of monoamine or folate metabolism. Only nonspecific changes were found. Secondary neurotransmitter abnormalities are frequently found in children with MDs (García-Cazorla et al., 2007; Tonduti et al., 2015; Burlina et al., 2017), and abnormal patterns suggestive of specific conditions other than primary defects have been recently described (Peall et al., 2017; Papandreou et al., 2018). Nevertheless, most of the commonly encountered secondary neurotransmitters abnormalities still lack a specific diagnostic value. This finding suggests that gene panel analysis has a limited diagnostic yield for the genetic diagnosis of inherited disorders of neurotransmission when biochemical studies are inconclusive. **Table 2** summarizes the relevant literature on the topic and shows the divisions in phenotypic groups of the different cohorts.

**Table 2:** Summary and comparison of published papers on genetic diagnosis of pediatric movement disorders. For each cohort, number of genes analyzed, sample size, genetic techniques and overall diagnostic yield are reported. All diagnostic categories of patients included in each cohort are also reported.

Study	Genes analyzed (No.)	Sample size (No.)	Technique(s)	Diagnostic Rate	DYT	PMD	Myoclonus	Chorea	NBIA	NT	Tremor	Hyperkinetic MD	Parkinsonism	Ataxia	HSP
Neveling et al. (2013)	151	50	WES	20%	X	–	–	–	–	–	–	–	–	X	X
van Egmond et al. (2017)	94	61	NGS panel	14%	X	–	–	–	–	–	–	–	–	–	–
Montaut et al. (2018)	127	378	NGS panel	22%	X	X	X	X	–	–		–	X	–	–
Reale et al. (2018)	65	221	NGS panel	11%	X	–	–	–	X	X	–	–	X	–	–
Cordeiro et al. (2018)	26	51	NGS, WES, Direct Seq	51%	X	–	–	X	–	–	X	X	–	–	–
Our cohort	102	148	NGS	28%	X	X	–	X	–	–	X	–	–	**–**	**–**

*WES, whole exome sequencing; NGS, next generation sequencing; DYT, dystonia; PMD, paroxysmal movement disorders; NBIA, neurodegeneration with brain iron accumulation; NT, inherited disorders of neurotransmitter metabolism; HSP, hereditary spastic paraplegias.*

With regard to the specific findings we encountered, gene panel analysis proved to be a valid method to expand the phenotypic and genotypic spectra of MD-associated genes, helping to expand the rapidly growing body of knowledge on recently discovered genes. Taken together, our results underline the extent to which the diagnostic yield of targeted gene panels for MD depends on the selection of patients and the investigation strategies adopted by each diagnostic facility. Nevertheless, targeted panels are a high-throughput sequencing strategy, which is efficient and cost-effective, with a good diagnostic rate. It is recommended that a multiple-strategy approach be adopted to improve the overall diagnostic rate for pediatric-onset MDs. This is necessary, considering the inability to detect copy number variants for these diseases, the low level of mosaicism, and the triplet-repeat expansions through NGS and the occurrence of disorders due to recently discovered or ultra-rare causative genes. A multiple-strategy diagnostic approach should combine target gene sequencing, (multiple) gene panel analysis, molecular cytogenetics, and whole exome or whole genome sequencing in a patient-tailored strategy (Cordeiro et al., 2018). However, the large number of patients with pediatric-onset MD and without a genetic diagnosis for all cohorts reported so far suggests the likely existence of several, still undiscovered, responsible genes (van Egmond et al., 2017; Cordeiro et al., 2018; Montaut et al., 2018; Papandreou et al., 2018; Reale et al., 2018).

From this perspective, gene panel analysis should be proposed as the first diagnostic tool for the genetic investigation of pediatric-onset MD, and the information obtained may be relevant for both diagnostic and research purposes.

## Data Availability Statement

The datasets generated for this study are available on request to the corresponding author.

## Ethics Statement

The studies involving human participants were reviewed and approved by Bambino Gesù Local Ethics Committee. Written informed consent to participate in this study was provided by the participants’ legal guardians or next of kin.

## Author Contributions

FG, GG, FS, and AC contributed to the conception and design of the study; FG and LB organized the database; FG, GG, and AC performed the statistical analysis; FG wrote the first draft of the manuscript; GG, FS, LB, and LT wrote sections of the manuscript; and FV, PC, EB, and AC revised the final draft. All authors contributed to the manuscript revision and read and approved the submitted version.

## Funding

This study was partially supported by the Ricerca Corrente funding from the Italian Ministry of Health to IRCCS Bambino Gesù.

accumulation; NGS, next generation sequencing; NT, inherited disorders of neurotransmitter metabolism; PKD, paroxysmal kinesigenic dyskinesia; PMD, paroxysmal movement disorders; XLD, X-linked; WES, whole exome sequencing

## Conflict of Interest

The authors declare that the research was conducted in the absence of any commercial or financial relationships that could be construed as a potential conflict of interest.

## Abbreviations

AD, autosomal dominant; AR, autosomal recessive; CSF: cerebrospinal fluid; DYT, dystonia; EA, episodic ataxia; EPD, exercise-induced paroxysmal dyskinesia; fHM, familial hemiplegic migraine; HSP, hereditary spastic paraplegias; MAF, minor allele frequency; MD, movement disorders; MRI, magnetic resonance imaging; NBIA, neurodegeneration with brain iron accumulation; NGS, next generation sequencing; NT, inherited disorders of neurotransmitter metabolism; PKD, paroxysmal kinesigenic dyskinesia; PMD, paroxysmal movement disorders; XLD, X-linked; WES, whole exome sequencing.
