# Supplementary material for: Diagnostic Yield of a Targeted Next-Generation Sequencing Gene Panel for Pediatric-Onset Movement Disorders: A 3-Year Cohort Study
Source: Front Genet. 2019 Oct 29;10:1026. doi: 10.3389/fgene.2019.01026 (PMC6828958; doi:10.3389/fgene.2019.01026)
Supplement: Supplementary file 1 [file Table_1.docx]

**Table 1**

| Overall **^1^**diagnostic Yield of Panel (N° mutated patients/total patients analyzed | Diagnostic Yield of the Panel considering only MD (N° mutated patients/total patients analyzed |
| --- | --- |
| 23% (46/204) | 28% (42/148) |

Legend: 1. The overall diagnostic yield, counting all the excluded patients; MD: movement disorders

From the original cohort, 38 patients were excluded because were referred with a clinical diagnosis of fHM (familial Hemiplegic Migraine) or paroxysmal torticollis and other 18 patient files were excluded due to incomplete chart documentation. The diagnostic rate in the fHM subgroup is 10% (4/38), particularly we detected 3 mutations in *CACNA1A* gene and 1 mutation in *ATP1A2* gene. .

**APPENDIX 2**

Complete list of genes included in the NEXTERA panel for Movement Disorders. Cytogenetics location, Phenotype description, MIM number and inheritance pattern are indicated. For genes that are not included in the MIM classification system or are included in the panel basing on a putative role in causing movement disorders, reference supporting their inclusion in the panel is provided.

| **Gene** | **Location** | **Phenotype** | **MIM number or reference** | **Inheritance** |
| --- | --- | --- | --- | --- |
| ***ADCY5*** | [3q21.1](https://www.omim.org/geneMap/3/551?start=-3&limit=10&highlight=551) | Dyskinesia, familial, with facial myokymia | [606703](https://www.omim.org/entry/606703) | AD |
| ***ADAR1*** | [1q21.3](https://www.omim.org/geneMap/1/1074?start=-3&limit=10&highlight=1074) | Aicardi-Goutieres syndrome 6 | [615010](https://www.omim.org/entry/615010) | AR |
|  |  | Dyschromatosis symmetrica hereditaria | [127400](https://www.omim.org/entry/127400) | AD |
| ***ADRA2B*** | [2q11.2](https://www.omim.org/geneMap/2/422?start=-3&limit=10&highlight=422) | Epilepsy, myoclonic, familial adult, 2 | [607876](https://www.omim.org/entry/607876) | AD |
| ***ANO3*** | [11p14.3-p14.2](https://www.omim.org/geneMap/11/235?start=-3&limit=10&highlight=235) | Dystonia 24 | [615034](https://www.omim.org/entry/615034) | AD |
| ***AR*** | [Xq12](https://www.omim.org/geneMap/X/365?start=-3&limit=10&highlight=365) | Spinal and bulbar muscular atrophy of Kennedy | [313200](https://www.omim.org/entry/313200) | XLR |
| ***ARFGEF2*** | [20q13.13](https://www.omim.org/geneMap/20/327?start=-3&limit=10&highlight=327) | Periventricular heterotopia with microcephaly | [608097](https://www.omim.org/entry/608097) | AR |
| ***ARX*** | [Xp21.3](https://www.omim.org/geneMap/X/128?start=-3&limit=10&highlight=128) | Epileptic encephalopathy, early infantile, 1 | [308350](https://www.omim.org/entry/308350) | XLR |
|  |  | Hydranencephaly with abnormal genitalia | [300215](https://www.omim.org/entry/300215) | XL |
|  |  | Lissencephaly, X-linked 2 | [300215](https://www.omim.org/entry/300215) | XL |
|  |  | Mental retardation, X-linked 29 and others | [300419](https://www.omim.org/entry/300419) | XLR |
|  |  | Partington syndrome | [309510](https://www.omim.org/entry/309510) | XLR |
|  |  | Proud syndrome | [300004](https://www.omim.org/entry/300004) | XL |
| ***ATP13A2*** | [1p36.13](https://www.omim.org/geneMap/1/184?start=-3&limit=10&highlight=184) | Kufor-Rakeb syndrome | [606693](https://www.omim.org/entry/606693) | AR |
|  |  | Spastic paraplegia 78, autosomal recessive | [617225](https://www.omim.org/entry/617225) | AR |
| ***ATP1A2*** | [1q23.2](https://www.omim.org/geneMap/1/1171?start=-3&limit=10&highlight=1171) | Alternating hemiplegia of childhood 1 | [104290](https://www.omim.org/entry/104290) | AD |
|  |  | Migraine, familial basilar | [602481](https://www.omim.org/entry/602481) | AD |
|  |  | Migraine, familial hemiplegic, 2 | [602481](https://www.omim.org/entry/602481) | AD |
| ***ATP1A3*** | [19q13.2](https://www.omim.org/geneMap/19/671?start=-3&limit=10&highlight=671) | Alternating hemiplegia of childhood 2 | [614820](https://www.omim.org/entry/614820) | AD |
|  |  | CAPOS syndrome | [601338](https://www.omim.org/entry/601338) | AD |
|  |  | Dystonia-12 | [128235](https://www.omim.org/entry/128235) | AD |
| ***ATP7B*** | [13q14.3](https://www.omim.org/geneMap/13/175?start=-3&limit=10&highlight=175) | Wilson disease | [277900](https://www.omim.org/entry/277900) | AR |
| ***C19orf12*** | [19q12](https://www.omim.org/geneMap/19/474?start=-3&limit=10&highlight=474) | Spastic paraplegia 43, autosomal recessive | [615043](https://www.omim.org/entry/615043) | AR |
|  |  | Neurodegeneration with brain iron accumulation 4 | [614298](https://www.omim.org/entry/614298) | AR |
| ***CACNA1A*** | [19p13.13](https://www.omim.org/geneMap/19/327?start=-3&limit=10&highlight=327) | Epileptic encephalopathy, early infantile, 42 | [617106](https://www.omim.org/entry/617106) | AD |
|  |  | Episodic ataxia, type 2 | [108500](https://www.omim.org/entry/108500) | AD |
|  |  | Migraine, familial hemiplegic, 1 | [141500](https://www.omim.org/entry/141500) | AD |
|  |  | Migraine, familial hemiplegic, 1, with progressive cerebellar ataxia | [141500](https://www.omim.org/entry/141500) | AD |
|  |  | Spinocerebellar ataxia 6 | [183086](https://www.omim.org/entry/183086) | AD |
| ***CACNA1B*** | [9q34.3](https://www.omim.org/geneMap/9/631?start=-3&limit=10&highlight=631) | Dystonia 23 | [614860](https://www.omim.org/entry/614860) | AD |
| ***CACNB4*** | [2q23.3](https://www.omim.org/geneMap/2/612?start=-3&limit=10&highlight=612) | Episodic ataxia, type 5 | [613855](https://www.omim.org/entry/613855) | AD |
|  |  | {Epilepsy, idiopathic generalized, susceptibility to, 9} | [607682](https://www.omim.org/entry/607682) | AD |
|  |  | {Epilepsy, juvenile myoclonic, susceptibility to, 6} | [607682](https://www.omim.org/entry/607682) | AD |
| ***CDKL5*** | [Xp22.13](https://www.omim.org/geneMap/X/98?start=-3&limit=10&highlight=98) | Epileptic encephalopathy, early infantile, 2 | [300672](https://www.omim.org/entry/300672) | XLD |
| ***CHD2*** | [15q26.1](https://www.omim.org/geneMap/15/475?start=-3&limit=10&highlight=475) | Epileptic encephalopathy, childhood-onset | [615369](https://www.omim.org/entry/615369) | AD |
| ***CIZ1*** | [9q34.11](https://www.omim.org/geneMap/9/482?start=-3&limit=10&highlight=482) | ?primary cervical dystonia | [1] | AD |
| ***CNTN2*** | [1q32.1](https://www.omim.org/geneMap/1/1454?start=-3&limit=10&highlight=1454) | ?Epilepsy, myoclonic, familial adult, 5 | [615400](https://www.omim.org/entry/615400) | AR |
| ***COASY*** | [17q21.2](https://www.omim.org/geneMap/17/541?start=-3&limit=10&highlight=541) | Neurodegeneration with brain iron accumulation 6 | [615643](https://www.omim.org/entry/615643) | AR |
|  |  | Pontocerebellar hypoplasia, type 12 | [618266](https://www.omim.org/entry/618266) | AR |
| ***COL6A3*** | [2q37.3](https://www.omim.org/geneMap/2/1019?start=-3&limit=10&highlight=1019) | Bethlem myopathy 1 | [158810](https://www.omim.org/entry/158810) | AD, AR |
|  |  | Dystonia 27 | [616411](https://www.omim.org/entry/616411) | AR |
|  |  | Ullrich congenital muscular dystrophy 1 | [254090](https://www.omim.org/entry/254090) | AD, AR |
| ***COMT*** | [22q11.21](https://www.omim.org/geneMap/22/50?start=-3&limit=10&highlight=50) | {Panic disorder, susceptibility to} | [167870](https://www.omim.org/entry/167870) | ?AD |
|  |  | {Schizophrenia, susceptibility to} | [181500](https://www.omim.org/entry/181500) | AD |
| ***CP*** | [3q24-q25](https://www.omim.org/geneMap/3/687?start=-3&limit=10&highlight=687) | Cerebellar ataxia | [604290](https://www.omim.org/entry/604290) | AR |
|  |  | Hemosiderosis, systemic, due to aceruloplasminemia | [604290](https://www.omim.org/entry/604290) | AR |
|  |  | [Hypoceruloplasminemia, hereditary] | [604290](https://www.omim.org/entry/604290) | AR |
| ***CSNK1D*** | [17q25.3](https://www.omim.org/geneMap/17/972?start=-3&limit=10&highlight=972) | Advanced sleep-phase syndrome, familial, 2 | [615224](https://www.omim.org/entry/615224) | AD |
| ***DBH*** | [9q34.2](https://www.omim.org/geneMap/9/557?start=-3&limit=10&highlight=557) | Orthostatic hypotension 1, due to DBH deficiency | [223360](https://www.omim.org/entry/223360) | AR |
| ***DDC*** | [7p12.2-p12.1](https://www.omim.org/geneMap/7/243?start=-3&limit=10&highlight=243) | Aromatic L-amino acid decarboxylase deficiency | [608643](https://www.omim.org/entry/608643) | AR |
| ***DNAJC6*** | [1p31.3](https://www.omim.org/geneMap/1/599?start=-3&limit=10&highlight=599) | Parkinson disease 19a, juvenile-onset | [615528](https://www.omim.org/entry/615528) | AR |
|  |  | Parkinson disease 19b, early-onset | [615528](https://www.omim.org/entry/615528) | AR |
| ***DNAL4*** | [22q13.1](https://www.omim.org/geneMap/22/256?start=-3&limit=10&highlight=256) | ?Mirror movements 3 | [616059](https://www.omim.org/entry/616059) | AR |
| ***FA2H*** | [16q23.1](https://www.omim.org/geneMap/16/583?start=-3&limit=10&highlight=583) | Spastic paraplegia 35, autosomal recessive | [612319](https://www.omim.org/entry/612319) | AR |
| ***FOXG1*** | [14q12](https://www.omim.org/geneMap/14/128?start=-3&limit=10&highlight=128) | Rett syndrome, congenital variant | [613454](https://www.omim.org/entry/613454) | AD |
| ***FTL*** | [19q13.33](https://www.omim.org/geneMap/19/828?start=-3&limit=10&highlight=828) | Hyperferritinemia-cataract syndrome | [600886](https://www.omim.org/entry/600886) | AD |
|  |  | L-ferritin deficiency, dominant and recessive | [615604](https://www.omim.org/entry/615604) | AD, AR |
|  |  | Neurodegeneration with brain iron accumulation 3 | [606159](https://www.omim.org/entry/606159) | AD |
| ***FBXO7*** | [22q12.3](https://www.omim.org/geneMap/22/187?start=-3&limit=10&highlight=187) | Parkinson disease 15, autosomal recessive | [260300](https://www.omim.org/entry/260300) | AR |
| ***GABRB2*** | [5q34](https://www.omim.org/geneMap/5/665?start=-3&limit=10&highlight=665) | Epileptic encephalopathy, infantile or early childhood, 2 | [617829](https://www.omim.org/entry/617829) | AD |
| ***GCH1*** | [14q22.2](https://www.omim.org/geneMap/14/227?start=-3&limit=10&highlight=227) | Dystonia, DOPA-responsive, with or without hyperphenylalaninemia | [128230](https://www.omim.org/entry/128230) | AD, AR |
|  |  | Hyperphenylalaninemia, BH4-deficient, B | [233910](https://www.omim.org/entry/233910) | AR |
| ***GNAL*** | [18p11.21](https://www.omim.org/geneMap/18/54?start=-3&limit=10&highlight=54) | Dystonia 25 | [615073](https://www.omim.org/entry/615073) | AD |
| ***GNAO1*** | [16q13](https://www.omim.org/geneMap/16/409?start=-3&limit=10&highlight=409) | Epileptic encephalopathy, early infantile, 17 | [615473](https://www.omim.org/entry/615473) | AD |
|  |  | Neurodevelopmental disorder with involuntary movements | [617493](https://www.omim.org/entry/617493) | AD |
| ***GRIN1*** | [9q34.3](https://www.omim.org/geneMap/9/613?start=-3&limit=10&highlight=613) | Neurodevelopmental disorder with or without hyperkinetic movements and seizures, autosomal dominant | [614254](https://www.omim.org/entry/614254) | AD |
|  |  | Neurodevelopmental disorder with or without hyperkinetic movements and seizures, autosomal recessive | [617820](https://www.omim.org/entry/617820) | AR |
| ***GTPBP2*** | [6p21.1](https://www.omim.org/geneMap/6/498?start=-3&limit=10&highlight=498) | Jaberi-Elahi syndrome | [617988](https://www.omim.org/entry/617988) | AR |
| ***KCNK18*** | [10q25.3](https://www.omim.org/geneMap/10/547?start=-3&limit=10&highlight=547) | {Migraine, with or without aura, susceptibility to, 13} | [613656](https://www.omim.org/entry/613656) |  |
| ***KCNA1*** | [12p13.32](https://www.omim.org/geneMap/12/38?start=-3&limit=10&highlight=38) | Episodic ataxia/myokymia syndrome | [160120](https://www.omim.org/entry/160120) | AD |
| ***KCNQ2*** | [20q13.33](https://www.omim.org/geneMap/20/418?start=-3&limit=10&highlight=418) | Epileptic encephalopathy, early infantile, 7 | [613720](https://www.omim.org/entry/613720) | AD |
|  |  | Myokymia | [121200](https://www.omim.org/entry/121200) | AD |
|  |  | Seizures, benign neonatal, 1 | [121200](https://www.omim.org/entry/121200) | AD |
| ***KCTD17*** | [22q12.3](https://www.omim.org/geneMap/22/216?start=-3&limit=10&highlight=216) | Dystonia 26, myoclonic | [616398](https://www.omim.org/entry/616398) | AD |
| ***KMT2B*** | [19q13.12](https://www.omim.org/geneMap/19/536?start=-3&limit=10&highlight=536) | Dystonia 28, childhood-onset | [617284](https://www.omim.org/entry/617284) | AD |
| ***MBIP*** | [14q13.3](https://www.omim.org/geneMap/14/156?start=-3&limit=10&highlight=156) | Putative role | [2] |  |
| ***MED20*** | [6p21.1](https://www.omim.org/geneMap/6/466?start=-3&limit=10&highlight=466) | ?infantile basal ganglia degeneration | [3] | AR |
| ***MR1*** | [2q35](https://www.omim.org/geneMap/2/883?start=-3&limit=10&highlight=883) | Paroxysmal nonkinesigenic dyskinesia 1 | [118800](https://www.omim.org/entry/118800) | AD |
| ***MRMV1 (DCC)*** | [18q21.2](https://www.omim.org/geneMap/18/177?start=-3&limit=10&highlight=177) | Mirror movements 1 and/or agenesis of the corpus callosum | [157600](https://www.omim.org/entry/157600) | AD |
| ***NKX2-1*** | [14q13.3](https://www.omim.org/geneMap/14/158?start=-3&limit=10&highlight=158) | Chorea, hereditary benign | [118700](https://www.omim.org/entry/118700) | AD |
|  |  | Choreoathetosis, hypothyroidism, and neonatal respiratory distress | [610978](https://www.omim.org/entry/610978) | AD |
|  |  | {Thyroid cancer, nonmedullary, 1} | [188550](https://www.omim.org/entry/188550) | AD |
| ***NUP62*** | [19q13.33](https://www.omim.org/geneMap/19/872?start=-3&limit=10&highlight=872) | Striatonigral degeneration, infantile | [271930](https://www.omim.org/entry/271930) | AR |
| ***PANK2*** | [20p13](https://www.omim.org/geneMap/20/58?start=-3&limit=10&highlight=58) | HARP syndrome | [607236](https://www.omim.org/entry/607236) | AR |
|  |  | Neurodegeneration with brain iron accumulation 1 | [234200](https://www.omim.org/entry/234200) | AR |
| ***PARK2*** | [6q26](https://www.omim.org/geneMap/6/936?start=-3&limit=10&highlight=936) | Adenocarcinoma of lung, somatic | [211980](https://www.omim.org/entry/211980) |  |
|  |  | Ovarian cancer, somatic | [167000](https://www.omim.org/entry/167000) |  |
|  |  | Parkinson disease, juvenile, type 2 | [600116](https://www.omim.org/entry/600116) | AR |
| ***PARK7*** | [1p36.23](https://www.omim.org/geneMap/1/103?start=-3&limit=10&highlight=103) | Parkinson disease 7, autosomal recessive early-onset | [606324](https://www.omim.org/entry/606324) | AR |
| ***PCBD1*** | [10q22.1](https://www.omim.org/geneMap/10/261?start=-3&limit=10&highlight=261) | Hyperphenylalaninemia, BH4-deficient, D | [264070](https://www.omim.org/entry/264070) | AR |
| ***PDE10A*** | [6q27](https://www.omim.org/geneMap/6/943?start=-3&limit=10&highlight=943) | Dyskinesia, limb and orofacial, infantile-onset | [616921](https://www.omim.org/entry/616921) | AR |
|  |  | Striatal degeneration, autosomal dominant | [616922](https://www.omim.org/entry/616922) | AD |
| ***PINK1*** | [1p36.12](https://www.omim.org/geneMap/1/218?start=-3&limit=10&highlight=218) | Parkinson disease 6, early onset | [605909](https://www.omim.org/entry/605909) | AR |
| ***PITX3*** | [10q24.32](https://www.omim.org/geneMap/10/473?start=-3&limit=10&highlight=473) | Anterior segment dysgenesis 1, multiple subtypes | [107250](https://www.omim.org/entry/107250) | AD |
|  |  | Cataract 11, multiple types | [610623](https://www.omim.org/entry/610623) | AD, AR |
|  |  | Cataract 11, syndromic, autosomal recessive | [610623](https://www.omim.org/entry/610623) | AD, AR |
| ***PLA2G6*** | [22q13.1](https://www.omim.org/geneMap/22/245?start=-3&limit=10&highlight=245) | Infantile neuroaxonal dystrophy 1 | [256600](https://www.omim.org/entry/256600) | AR |
|  |  | Neurodegeneration with brain iron accumulation 2B | [610217](https://www.omim.org/entry/610217) | AR |
|  |  | Parkinson disease 14, autosomal recessive | [612953](https://www.omim.org/entry/612953) | AR |
| ***PNMT*** | [17q12](https://www.omim.org/geneMap/17/455?start=-3&limit=10&highlight=455) | Putative role | [4] |  |
| ***PNPO*** | [17q21.32](https://www.omim.org/geneMap/17/634?start=-3&limit=10&highlight=634) | Pyridoxamine 5'-phosphate oxidase deficiency | [610090](https://www.omim.org/entry/610090) | AR |
| ***POC1B*** | [12q21.33](https://www.omim.org/geneMap/12/615?start=-3&limit=10&highlight=615) | Cone-rod dystrophy 20 | [615973](https://www.omim.org/entry/615973) | AR |
| ***PRKRA*** | [2q31.2](https://www.omim.org/geneMap/2/726?start=-3&limit=10&highlight=726) | Dystonia 16 | [612067](https://www.omim.org/entry/612067) | AR |
| ***PRRT2*** | [16p11.2](https://www.omim.org/geneMap/16/301?start=-3&limit=10&highlight=301) | Convulsions, familial infantile, with paroxysmal choreoathetosis | [602066](https://www.omim.org/entry/602066) | AD |
|  |  | Episodic kinesigenic dyskinesia 1 | [128200](https://www.omim.org/entry/128200) | AD |
|  |  | Seizures, benign familial infantile, 2 | [605751](https://www.omim.org/entry/605751) | AD |
| ***PTS*** | [11q23.1](https://www.omim.org/geneMap/11/855?start=-3&limit=10&highlight=855) | Hyperphenylalaninemia, BH4-deficient, A | [261640](https://www.omim.org/entry/261640) | AR |
| ***QDPR*** | [4p15.32](https://www.omim.org/geneMap/4/108?start=-3&limit=10&highlight=108) | Hyperphenylalaninemia, BH4-deficient, C | [261630](https://www.omim.org/entry/261630) | AR |
| ***RAD51A*** | [15q15.1](https://www.omim.org/geneMap/15/97?start=-3&limit=10&highlight=97) | ?Fanconi anemia, complementation group R | [617244](https://www.omim.org/entry/617244) | AD |
|  |  | Mirror movements 2 | [614508](https://www.omim.org/entry/614508) | AD |
|  |  | {Breast cancer, susceptibility to} | [114480](https://www.omim.org/entry/114480) | AD, SMu |
| ***RELN*** | [7q22.1](https://www.omim.org/geneMap/7/517?start=-3&limit=10&highlight=517) | Lissencephaly 2 (Norman-Roberts type) | [257320](https://www.omim.org/entry/257320) | AR |
|  |  | {Epilepsy, familial temporal lobe, 7} | [616436](https://www.omim.org/entry/616436) | AD |
| ***SLC39A8*** | [4q24](https://www.omim.org/geneMap/4/404?start=-3&limit=10&highlight=404) | Congenital disorder of glycosylation, type IIn | [616721](https://www.omim.org/entry/616721) | AR |
| ***SCN1A*** | [2q24.3](https://www.omim.org/geneMap/2/659?start=-3&limit=10&highlight=659) | Epilepsy, generalized, with febrile seizures plus, type 2 | [604403](https://www.omim.org/entry/604403) | AD |
|  |  | Epileptic encephalopathy, early infantile, 6 (Dravet syndrome) | [607208](https://www.omim.org/entry/607208) | AD |
|  |  | Febrile seizures, familial, 3A | [604403](https://www.omim.org/entry/604403) | AD |
|  |  | Migraine, familial hemiplegic, 3 | [609634](https://www.omim.org/entry/609634) | AD |
| ***SCN2A*** | [2q24.3](https://www.omim.org/geneMap/2/656?start=-3&limit=10&highlight=656) | Epileptic encephalopathy, early infantile, 11 | [613721](https://www.omim.org/entry/613721) | AD |
|  |  | Seizures, benign familial infantile, 3 | [607745](https://www.omim.org/entry/607745) | AD |
| ***SCN8A*** | [12q13.13](https://www.omim.org/geneMap/12/358?start=-3&limit=10&highlight=358) | ?Cognitive impairment with or without cerebellar ataxia | [614306](https://www.omim.org/entry/614306) | AD |
|  |  | ?Myoclonus, familial, 2, | [618364](https://www.omim.org/entry/618364) |  |
|  |  | Epileptic encephalopathy, early infantile, 13 | [614558](https://www.omim.org/entry/614558) | AD |
|  |  | Seizures, benign familial infantile, 5 | [617080](https://www.omim.org/entry/617080) | AD |
| ***SETD5*** | [3p25.3](https://www.omim.org/geneMap/3/30?start=-3&limit=10&highlight=30) | Mental retardation, autosomal dominant 23 | [615761](https://www.omim.org/entry/615761) | AD |
| ***SGCE*** | [7q21.3](https://www.omim.org/geneMap/7/399?start=-3&limit=10&highlight=399) | Dystonia-11, myoclonic | [159900](https://www.omim.org/entry/159900) | AD |
| ***SLC16A2*** | [Xq13.2](https://www.omim.org/geneMap/X/415?start=-3&limit=10&highlight=415) | Allan-Herndon-Dudley syndrome | [300523](https://www.omim.org/entry/300523) | XL |
| ***SLC18A2*** | [10q25.3](https://www.omim.org/geneMap/10/548?start=-3&limit=10&highlight=548) | ?Parkinsonism-dystonia, infantile, 2 | [618049](https://www.omim.org/entry/618049) | AR |
| ***SLC19A3*** | [2q36.3](https://www.omim.org/geneMap/2/949?start=-3&limit=10&highlight=949) | Thiamine metabolism dysfunction syndrome 2 (biotin- or thiamine-responsive encephalopathy type 2) | [607483](https://www.omim.org/entry/607483) | AR |
| ***SLC1A3*** | [5p13.2](https://www.omim.org/geneMap/5/107?start=-3&limit=10&highlight=107) | Episodic ataxia, type 6 | [612656](https://www.omim.org/entry/612656) | AD |
| ***SLC25A19*** | [17q25.1](https://www.omim.org/geneMap/17/863?start=-3&limit=10&highlight=863) | Microcephaly, Amish type | [607196](https://www.omim.org/entry/607196) | AR |
|  |  | Thiamine metabolism dysfunction syndrome 4 (progressive polyneuropathy type) | [613710](https://www.omim.org/entry/613710) | AR |
| ***SLC2A1*** | [1p34.2](https://www.omim.org/geneMap/1/454?start=-3&limit=10&highlight=454) | Dystonia 9 | [601042](https://www.omim.org/entry/601042) | AD |
|  |  | GLUT1 deficiency syndrome 1, infantile onset, severe | [606777](https://www.omim.org/entry/606777) | AD, AR |
|  |  | GLUT1 deficiency syndrome 2, childhood onset | [612126](https://www.omim.org/entry/612126) | AD |
|  |  | Stomatin-deficient cryohydrocytosis with neurologic defects | [608885](https://www.omim.org/entry/608885) | AD |
|  |  | {Epilepsy, idiopathic generalized, susceptibility to, 12} | [614847](https://www.omim.org/entry/614847) | AD |
| ***SLC30A10*** | [1q41](https://www.omim.org/geneMap/1/1542?start=-3&limit=10&highlight=1542) | Hypermanganesemia with dystonia 1 | [613280](https://www.omim.org/entry/613280) | AR |
| ***SLC39A14*** | [8p21.3](https://www.omim.org/geneMap/8/106?start=-3&limit=10&highlight=106) | ?Hyperostosis cranalis interna | [144755](https://www.omim.org/entry/144755) | AD |
|  |  | Hypermanganesemia with dystonia 2 | [617013](https://www.omim.org/entry/617013) | AR |
| ***SLC5A1*** | [22q12.3](https://www.omim.org/geneMap/22/181?start=-3&limit=10&highlight=181) | Glucose/galactose malabsorption | [606824](https://www.omim.org/entry/606824) | AR |
| ***SLC6A3*** | [5p15.33](https://www.omim.org/geneMap/5/23?start=-3&limit=10&highlight=23) | Parkinsonism-dystonia, infantile, 1 | [613135](https://www.omim.org/entry/613135) | AR |
|  |  | {Nicotine dependence, protection against} | [188890](https://www.omim.org/entry/188890) |  |
| ***SPR*** | [2p13.2](https://www.omim.org/geneMap/2/320?start=-3&limit=10&highlight=320) | Dystonia, dopa-responsive, due to sepiapterin reductase deficiency | [612716](https://www.omim.org/entry/612716) | AR, ?AD |
| ***STXBP1*** | [9q34.11](https://www.omim.org/geneMap/9/466?start=-3&limit=10&highlight=466) | Epileptic encephalopathy, early infantile, 4 | [612164](https://www.omim.org/entry/612164) | AD |
| ***SYNJ1*** | [21q22.11](https://www.omim.org/geneMap/21/59?start=-3&limit=10&highlight=59) | Epileptic encephalopathy, early infantile, 53 | [617389](https://www.omim.org/entry/617389) | AR |
|  |  | Parkinson disease 20, early-onset | [615530](https://www.omim.org/entry/615530) | AR |
| ***SYT1*** | [12q21.2](https://www.omim.org/geneMap/12/593?start=-3&limit=10&highlight=593) | Baker-Gordon syndrome | [618218](https://www.omim.org/entry/618218) | AD |
| ***TAF1*** | [Xq13.1](https://www.omim.org/geneMap/X/395?start=-3&limit=10&highlight=395) | Dystonia-Parkinsonism, X-linked | [314250](https://www.omim.org/entry/314250) | XLR |
|  |  | Mental retardation, X-linked, syndromic 33 | [300966](https://www.omim.org/entry/300966) | XLR |
| ***TBC1D24*** | [16p13.3](https://www.omim.org/geneMap/16/97?start=-3&limit=10&highlight=97) | Deafness , autosomal recessive 86 | [614617](https://www.omim.org/entry/614617) | AR |
|  |  | Deafness, autosomal dominant 65 | [616044](https://www.omim.org/entry/616044) | AD |
|  |  | DOORS syndrome | [220500](https://www.omim.org/entry/220500) | AR |
|  |  | Epileptic encephalopathy, early infantile, 16 | [615338](https://www.omim.org/entry/615338) | AR |
|  |  | Myoclonic epilepsy, infantile, familial | [605021](https://www.omim.org/entry/605021) | AR |
| ***TBCD*** | [17q25.3](https://www.omim.org/geneMap/17/981?start=-3&limit=10&highlight=981) | Encephalopathy, progressive, early-onset, with brain atrophy and thin corpus callosum | [617193](https://www.omim.org/entry/617193) | AR |
| ***TBCE*** | [1q42.3](https://www.omim.org/geneMap/1/1635?start=-3&limit=10&highlight=1635) | Encephalopathy, progressive, with amyotrophy and optic atrophy | [617207](https://www.omim.org/entry/617207) | AR |
|  |  | Hypoparathyroidism-retardation-dysmorphism syndrome | [241410](https://www.omim.org/entry/241410) | AR |
|  |  | Kenny-Caffey syndrome, type 1 | [244460](https://www.omim.org/entry/244460) | AR |
| ***TBCK*** | [4q24](https://www.omim.org/geneMap/4/419?start=-3&limit=10&highlight=419) | Hypotonia, infantile, with psychomotor retardation and characteristic facies 3 | [616900](https://www.omim.org/entry/616900) | AR |
| ***TBL1XR1*** | [3q26.32](https://www.omim.org/geneMap/3/786?start=-3&limit=10&highlight=786) | Mental retardation, autosomal dominant 41 | [616944](https://www.omim.org/entry/616944) | AD |
|  |  | Pierpont syndrome | [602342](https://www.omim.org/entry/602342) | AD |
| ***TH*** | [11p15.5](https://www.omim.org/geneMap/11/72?start=-3&limit=10&highlight=72) | Segawa syndrome, recessive | [605407](https://www.omim.org/entry/605407) | AR |
| ***THAP1*** | [8p11.21](https://www.omim.org/geneMap/8/220?start=-3&limit=10&highlight=220) | Dystonia 6, torsion | [602629](https://www.omim.org/entry/602629) | AD |
| ***TOR1A*** | [9q34.11](https://www.omim.org/geneMap/9/514?start=-3&limit=10&highlight=514) | Dystonia-1, torsion | [128100](https://www.omim.org/entry/128100) | AD |
|  |  | {Dystonia-1, modifier of} |  |  |
| ***TPH1*** | [11p15.1](https://www.omim.org/geneMap/11/206?start=-3&limit=10&highlight=206) | Putative role | [5] |  |
| ***TPH2*** | [12q21.1](https://www.omim.org/geneMap/12/576?start=-3&limit=10&highlight=576) | {Attention deficit-hyperactivity disorder, susceptibility to, 7} | [613003](https://www.omim.org/entry/613003) |  |
|  |  | {Unipolar depression, susceptibility to} | [608516](https://www.omim.org/entry/608516) |  |
| ***TUBB4A*** | [19p13.3](https://www.omim.org/geneMap/19/168?start=-3&limit=10&highlight=168) | Dystonia 4, torsion, autosomal dominant | [128101](https://www.omim.org/entry/128101) | AD |
|  |  | Leukodystrophy, hypomyelinating, 6 | [612438](https://www.omim.org/entry/612438) | AD |
| ***VAC14*** | [16q22.1-q22.2](https://www.omim.org/geneMap/16/554?start=-3&limit=10&highlight=554) | Striatonigral degeneration, childhood-onset | [617054](https://www.omim.org/entry/617054) | AR |
| ***VPS16*** | [20p13](https://www.omim.org/geneMap/20/37?start=-3&limit=10&highlight=37) | ?autosomal recessive adolescent-onset primary dystonia | [6] | AR |
| ***WDR45*** | [Xp11.23](https://www.omim.org/geneMap/X/260?start=-3&limit=10&highlight=260) | Neurodegeneration with brain iron accumulation 5 | [300894](https://www.omim.org/entry/300894) | XLD |

1. Xiao J, Uitti RJ, Zhao Y, et al (2012) Mutations in CIZ1 cause adult onset primary cervical dystonia. Ann Neurol 71:458–469. https://doi.org/10.1002/ana.23547

2. Invernizzi F, Zorzi G, Legati A, et al (2018) Benign hereditary chorea and deletions outside NKX2-1: What’s the role of MBIP? Eur J Med Genet 61:581–584. https://doi.org/10.1016/j.ejmg.2018.03.011

3. Vodopiutz J, Schmook MT, Konstantopoulou V, et al (2015) MED20 mutation associated with infantile basal ganglia degeneration and brain atrophy. Eur J Pediatr 174:113–118. https://doi.org/10.1007/s00431-014-2463-7

4. Gearhart DA, Neafsey EJ, Collins MA (2002) Phenylethanolamine N-methyltransferase has beta-carboline 2N-methyltransferase activity: hypothetical relevance to Parkinson’s disease. Neurochem Int 40:611–620

5. Calvo AC, Scherer T, Pey AL, et al (2010) Effect of pharmacological chaperones on brain tyrosine hydroxylase and tryptophan hydroxylase 2. J Neurochem 114:853–863. https://doi.org/10.1111/j.1471-4159.2010.06821.x

6. Cai X, Chen X, Wu S, et al (2016) Homozygous mutation of VPS16 gene is responsible for an autosomal recessive adolescent-onset primary dystonia. Sci Rep 6:25834. https://doi.org/10.1038/srep25834
